# A Modular Strategy to Engineer Complex Tissues and Organs

**DOI:** 10.1002/advs.201700402

**Published:** 2018-02-14

**Authors:** Anna D. Dikina, Daniel S. Alt, Samuel Herberg, Alexandra McMillan, Hannah A. Strobel, Zijie Zheng, Meng Cao, Bradley P. Lai, Oju Jeon, Victoria Ivy Petsinger, Calvin U. Cotton, Marsha W. Rolle, Eben Alsberg

**Affiliations:** ^1^ Department of Biomedical Engineering Case Western Reserve University 10900 Euclid Ave Cleveland OH 44106 USA; ^2^ Department of Pathology Case Western Reserve University 10900 Euclid Ave Cleveland OH 44106 USA; ^3^ Department of Biomedical Engineering Worcester Polytechnic Institute 100 Institute Road Worcester MA 01609 USA; ^4^ Department of Pediatrics Department of Physiology and Biophysics Case Western Reserve University 10900 Euclid Ave Cleveland OH 44106 USA; ^5^ Department of Orthopaedic Surgery National Center for Regenerative Medicine Case Western Reserve University 10900 Euclid Ave Cleveland OH 44106 USA

**Keywords:** composite tissue, microparticle, regenerative medicine, tissue engineering, trachea

## Abstract

Currently, there are no synthetic or biologic materials suitable for long‐term treatment of large tracheal defects. A successful tracheal replacement must (1) have radial rigidity to prevent airway collapse during respiration, (2) contain an immunoprotective respiratory epithelium, and (3) integrate with the host vasculature to support epithelium viability. Herein, biopolymer microspheres are used to deliver chondrogenic growth factors to human mesenchymal stem cells (hMSCs) seeded in a custom mold that self‐assemble into cartilage rings, which can be fused into tubes. These rings and tubes can be fabricated with tunable wall thicknesses and lumen diameters with promising mechanical properties for airway collapse prevention. Epithelialized cartilage is developed by establishing a spatially defined composite tissue composed of human epithelial cells on the surface of an hMSC‐derived cartilage sheet. Prevascular rings comprised of human umbilical vein endothelial cells and hMSCs are fused with cartilage rings to form prevascular–cartilage composite tubes, which are then coated with human epithelial cells, forming a tri‐tissue construct. When prevascular– cartilage tubes are implanted subcutaneously in mice, the prevascular structures anastomose with host vasculature, demonstrated by their ability to be perfused. This microparticle–cell self‐assembly strategy is promising for engineering complex tissues such as a multi‐tissue composite trachea.

## 1 Introduction

Patients suffering from tracheal stenosis have a significantly reduced quality of life. The diseased region of the trachea cannot be resected when more than half of the airway is affected in adults.^1^ Thus, tracheal tissue engineering has the exciting potential to fill this gap and will have a tremendous impact for the patients in need. A variety of tracheal replacement strategies have been developed in vitro and implanted in vivo, both in animals and human patients, including cell‐free artificial prostheses,^2^ autografts,^3^ intact allografts or decellularized allografts often seeded with the recipient's own cells,^4^ high‐cell density constructs derived from mature cell sources,^5^ and primarily scaffold‐based tissue engineered constructs.^6^ Despite the broad range of approaches, each has its own shortcomings ranging from the technical, such as restenosis,[[qv: 1c,3b]] to the practical, such as lack of tissue availability.

The adult trachea has 18–22 tracheal cartilage rings, which keep the airway patent during respiration.[[qv: 1a]] The tracheal lumen is epithelialized with a ciliated mucosa which acts as a protective barrier, humidifies inspired air and clears secretions from the lungs.[[qv: 1a]] A vascularized elastic tissue encases the cartilaginous rings and houses blood vessels that provide nutrients and oxygen to tracheal tissues.[[qv: 1a]] Keeping these attributes in mind, a functional tracheal replacement must therefore meet critical design criteria that include: (1) having radial rigidity, (2) possessing a luminal epithelium, and (3) supporting neovascularization to restore an open and functional airway while avoiding restenosis, bacterial infections, and ischemic necrosis.^7^ A modular tracheal engineering approach that employs scaffold‐free 3D tissue building blocks is an attractive option to address these three functional requirements. The ultimate goal is to fuse three types of engineered tissues into a continuous tracheal replacement by employing a custom‐built culture system, coupled with localized bioactive factor delivery, to provide spatial and temporal control over tissue formation.

To address the requirement for mechanical integrity of the engineered trachea, our group recently demonstrated an approach for the production of stiff, human mesenchymal stem cell (hMSC)‐derived cartilage tubes by fusing cartilaginous rings in a custom culture system.^8^ A key feature of this system is the incorporation of growth factor releasing gelatin microspheres that allow for controlled chondrogenic signal presentation to drive cartilage formation. In this work, engineered cartilaginous rings and tubes were shown to have mechanical properties similar or better than comparably sized native rat trachea segments.^8^ This study demonstrates that the engineered tissues can be formed in clinically relevant specific geometries and sizes that match human tissue dimensions by modulating the thickness and diameter of the engineered cartilage rings and tubes. To address the second element of a mature engineered trachea, an epithelium must be able to be cultured on the luminal surface of the construct.[[qv: 1c]] To that end, we use a human bronchial epithelial cell line (hBECs) to engineer an epithelial—cartilaginous bilayer as proof‐of‐principle to demonstrate that the engineered cartilage tissue can support an epithelial lining. Epithelialized cartilage bilayers were cultured either submerged in medium or at an air–liquid interface (ALI), which has been shown in previous studies to be beneficial for respiratory epithelial maturation.^9^ Next, vascularization is essential for the success of any tissue engineered construct and is currently a major limitation in the field of tissue engineering. The diffusional limitation of oxygen requires all metabolically active cells within the body to reside within ≈150–200 µm of a capillary,^10^ thus necessitating the microvascularization of thick tissue engineered constructs such as a trachea. Guiding the tissue engineered construct through the initial phases of vasculogenesis might accelerate neovascularization and anastomosis with host vasculature upon implantation.^11^ Human umbilical vein endothelial cells (HUVECs) have been cocultured with various stromal supporting cells for vasculogenic purposes.^12^ Here, HUVECs were cocultured with hMSCs in vasculogenic media to generate prevascular tissue rings, where the cells self‐assembled into early microvascular structures. These were then fused with cartilage rings to form prevascular—cartilage composite tissue tubes. Finally, these composite tissues were (1) seeded with human tracheal epithelial cells (HTEs) to engineer a tri‐tissue construct with spatial control of tissue composition and phenotype and (2) implanted subcutaneously in mice to demonstrate the ability of the tissues to anastomose with host vasculature.

**Figure 1:**
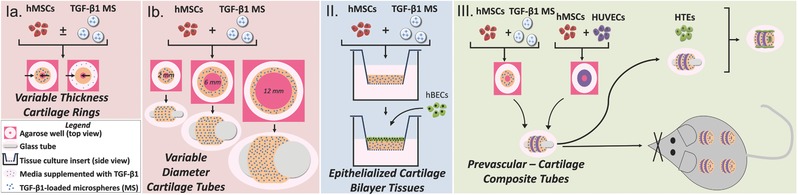
Schematic overview of the cartilage (Part Ia and Ib), epithelial–cartilage (Part II) and prevascular–cartilage and tri‐tissue (Part III) engineering components of this work aimed toward the generation of a multi‐tissue tracheal construct for airway repair.

The system presented herein has the potential to allow for the fabrication of many different complex, vascularized tubular tissues and organs. By adjusting the composition of the individual tissue rings, structures such as blood vessels, gastrointestinal tract, and ureters may be developed. This paper describes the application of this base technology to respiratory airway engineering for functional tracheal replacement. A schematic depicting fabrication of each of these tissues is presented in **Figure** **1**.

## 2 Results

### 2.1 Part Ia—Cartilage Rings with Defined Wall Thickness

#### 2.1.1 Macroscopic and Histological Assessment

**Figure 2:**
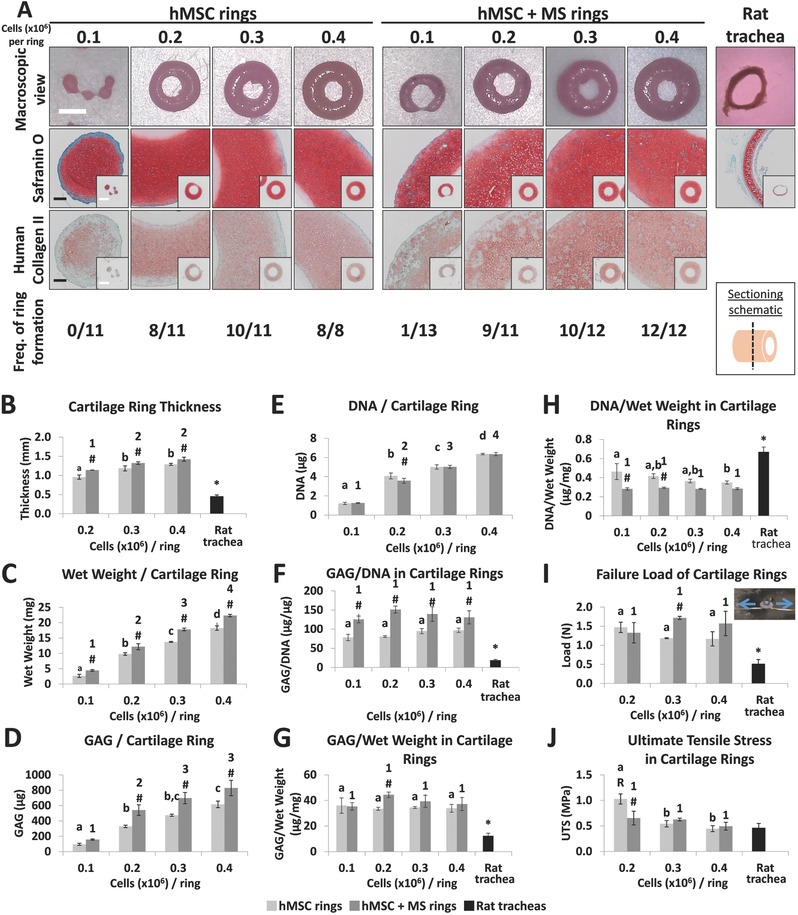
Analysis of tissue engineered cartilage rings formed with different cell numbers. A) Macroscopic images of rings were acquired, GAGs were stained with Safranin O (pink/red), immunohistochemistry was performed for human type II collagen (red) and the frequency of tissue engineered cartilage ring formation with varied cell number were reported compared to rat trachea. MS, microspheres; Fast Green counterstain (blue/green). Black scale bars are 200 µm; white scale bars are 2 mm. Images in a single row are the same magnification. B) Ring thickness was measured in tissue engineered hMSC (light gray) and hMSC + MS (dark gray) cartilage rings (*N* = 3) and compared to rat tracheal segments (black, *N* = 4). C) Construct wet weight, D) GAG content, E) DNA content, F) GAG normalized to DNA, G) GAG normalized to tissue wet weight and H) DNA normalized to tissue wet weight were acquired from cartilage rings (*N* = 3–4) and, for (F), (G) and (H), rat tracheal segments (*N* = 4). I) Failure load during pull‐to‐failure uniaxial testing (pictured in I, inset) and J) ultimate tensile strength (UTS; load normalized by area) were measured in engineered rings (*N* = 3) and compared to rat tracheal segments (black, *N* = 4). MS = microspheres. a,b,c,d: hMSC groups without common letter differ (*p* < 0.05); 1,2,3,4: hMSC + MS groups without common number differ (*p* < 0.05); #: significantly different than hMSC group (*p* < 0.05); *: rat trachea is significantly different compared to all other groups (*p* < 0.05); R: significantly different than rat trachea (*p* < 0.05). Data shown as mean ± SD.

With the goal of controlling the thickness of cartilage rings, the number of hMSCs used to form the rings was varied from 0.1 to 0.4 million cells per ring, and TGF‐β1 was presented either in the media or from incorporated microspheres (MS). Rings assembled from a greater number of hMSCs had a higher frequency of ring formation, were grossly (**Figure** **2**A) and quantitatively thicker (Figure 2B) and were heavier (Figure 2C). Ring thickness could be modulated by 25–35% by increasing cell number. Samples that did not form rings resulted in C‐shaped constructs or 1–3 individual aggregates. Microsphere‐containing rings were significantly thicker than hMSC‐only constructs and all engineered rings were thicker than cartilage ring segments from rat tracheas. Glycosaminoglycan (GAG; a prevalent polysaccharide in hyaline cartilage) staining was strong in all engineered tissues and appeared similar to staining in rat tracheal sections (Figure 2A). Type II collagen, the main collagenous component of hyaline cartilage ECM, was also apparent in both types of engineered cartilage (Figure 2A). Compared to hMSC‐only constructs, hMSC + MS rings appeared to have less of a fibrous capsule on the outer edge of the tissue.

#### 2.1.2 Biochemical Analysis

By measuring the amount of GAG and DNA in the variable thickness cartilage rings, quantitative differences in tissue composition as a function of the number of cells used during fabrication were elucidated (Figure 2C–H). Rings composed of more cells contained more GAG (Figure 2D) and DNA (Figure 2E). Chondrogenesis was not affected by cell number as the GAG/DNA (Figure 2F) and GAG/wet weight (Figure 2G) values were the same in rings made with varying cell numbers. As expected, hMSC + MS rings weighed more (Figure 2C) and contained significantly more GAG (Figure 2D, except 0.1 million cells per ring) and GAG/DNA (Figure 2F) than hMSC‐only rings. Compared to rat tracheal segments, engineered tissues had significantly more GAG/DNA (Figure 2F), more GAG/wet weight (Figure 2G), and less DNA/wet weight (Figure 2H). This was anticipated because the tracheal segments are composed of other tissues in addition to cartilage.

#### 2.1.3 Mechanical Analysis

All engineered rings required a significantly greater load to rupture under uniaxial tension compared to rat tracheal rings (1.21 ± 0.16 mm vertically) (Figure 2I), demonstrating biomechanical functionality and potential ability to maintain tracheal luminal patency. In addition, the ultimate tensile stress (UTS, failure load normalized by loaded area) was at least as high as that of the rat trachea in all groups (Figure 2J) signifying that the engineered cartilage tissue is at least as strong as the native rat tracheal tissue. Similar failure loads and UTS values across the different thickness rings (except the UTS of 0.2 million cells per ring without microspheres) indicate that geometry does not affect the mechanical properties of the engineered cartilage for the range of wall thickness examined.

### 2.2 Part Ib—Cartilage Tubes with Defined Lumen Diameters

#### 2.2.1 Macroscopic and Histological Assessment

**Figure 3:**
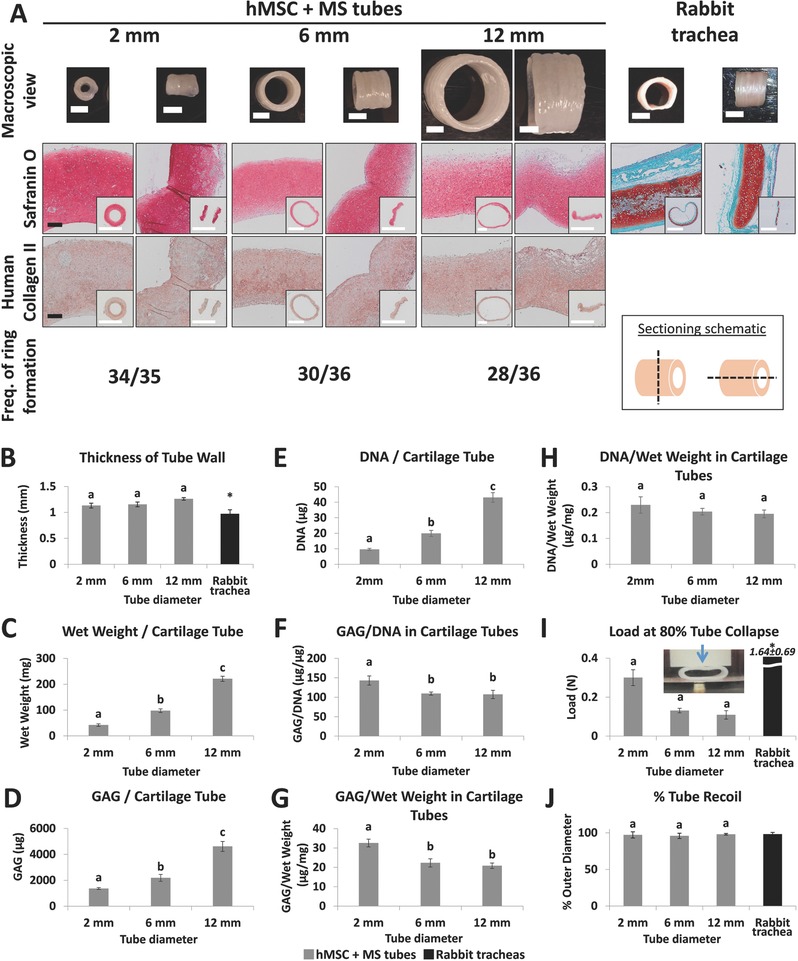
Macroscopic and histological images, and biochemical and mechanical analysis of cartilage tubes of 2, 6, and 12 mm inner diameters and the frequency of formation of tissue engineered rings used to make cartilage tubes. A) Macroscopic images of tubes were acquired, GAGs were stained with Safranin O (pink/red), immunohistochemistry was performed for human type II collagen (red) and the frequency of ring formation of 2, 6, and 12 mm inner diameter cartilage tubes was recorded. Tissue tubes were cut to show transverse and longitudinal sections. MS, microspheres; Fast Green counterstain (blue/green). Black scale bars are 200 µm; white scale bars are 4 mm. Images without scale bars in a single row are the same magnification. B) Tube wall thickness was measured in five‐ring engineered cartilage tubes (gray, *N* = 3) and compared to rabbit tracheal segments (black, *N* = 6). C) Construct wet weight, D) GAG content, E) DNA content, F) GAG normalized to DNA, G) GAG normalized to tissue wet weight, and H) DNA normalized to tissue wet weight were acquired from tissue engineered two‐ring cartilage tubes (*N* = 4–5). I) Load required to collapse 80% of the five‐ring engineered tube lumen diameter and J) tube recoil after luminal compression (I inset) were measured (*N* = 3) and compared to rabbit tracheal segments (black, *N* = 6). MS = microspheres. a,b,c: hMSC + MS groups without common letter differ (*p* < 0.05); *: rabbit trachea is significantly different compared to all other groups (*p* < 0.05). MS, microspheres. Data shown as mean ± SD.

To demonstrate the scalability of this system, cartilaginous rings comprised of hMSCs and TGF‐β1‐microspheres were fabricated with three different inner diameters and then fused into cartilage tubes with defined lumen dimensions. Rings assembled in larger diameter wells had a lower frequency of ring formation even though the cell and microsphere numbers were linearly scaled with the diameter of the wells (**Figure** **3**A). Cartilage rings of each diameter successfully fused into firm cartilage tubes. The lumen of the tubes was smooth while the outer wall was ribbed. Rabbit tracheal sections resembled the dimensions and gross morphology of 6 mm engineered cartilage tubes. The wall thickness of the 2, 6, and 12 mm diameter engineered cartilage tubes was not affected by the lumen size (Figure 3B). Additionally, 6 mm cartilage tube walls were only 16% thicker than rabbit tracheal walls, which had similar lumen diameters. GAG staining was strong in all engineered tubes but appeared weaker than staining in rabbit tracheal sections (Figure 3A). Type II collagen stained uniformly with strong intensity in the engineered cartilage tubes (Figure 3A).

#### 2.2.2 Biochemical Analysis

Biochemical evaluation of cartilage tubes (Figure 3C–H) revealed that larger diameter tubes were heavier (Figure 3C) and contained more GAG (Figure 3D) and DNA (Figure 3E). Interestingly, tubes with larger lumen diameter (6 and 12 mm) had decreased GAG/DNA (Figure 3F) and GAG/wet weight (Figure 3G) compared to 2 mm lumen diameter tubes while maintaining similar amounts of DNA normalized to wet weight (Figure 3H) indicating somewhat decreased chondrogenesis in the larger tubes.

#### 2.2.3 Mechanical Analysis

To evaluate the biomechanical properties of engineered cartilage tubes and compare their behavior to native rabbit tracheas, the lumen of each tube or trachea was compressed by applying a perpendicular load (Movies S1–S3, Supporting Information, correspond to the testing of 2, 6, and 12 mm inner diameter tubes, respectively). All engineered tubes required a significantly smaller load to collapse 80% of the lumen diameter compared to rabbit tracheal segments of similar length (Figure 3I). The load at 80% collapse was significantly larger for 2 mm diameter tubes compared to 6 and 12 mm tubes. Subsequently, the compressive load was removed and the outer diameter of each tube before and after collapse was compared to calculate the recoil of engineered tubes and rabbit tracheas. All luminally collapsed tubes recoiled to nearly 100% of the original outer diameter with no significant differences between the groups (Figure 3J).

### 2.3 Part II—Epithelialized Cartilage Bilayers

#### 2.3.1 Histological Assessment and Immunohistochemistry

**Figure 4:**
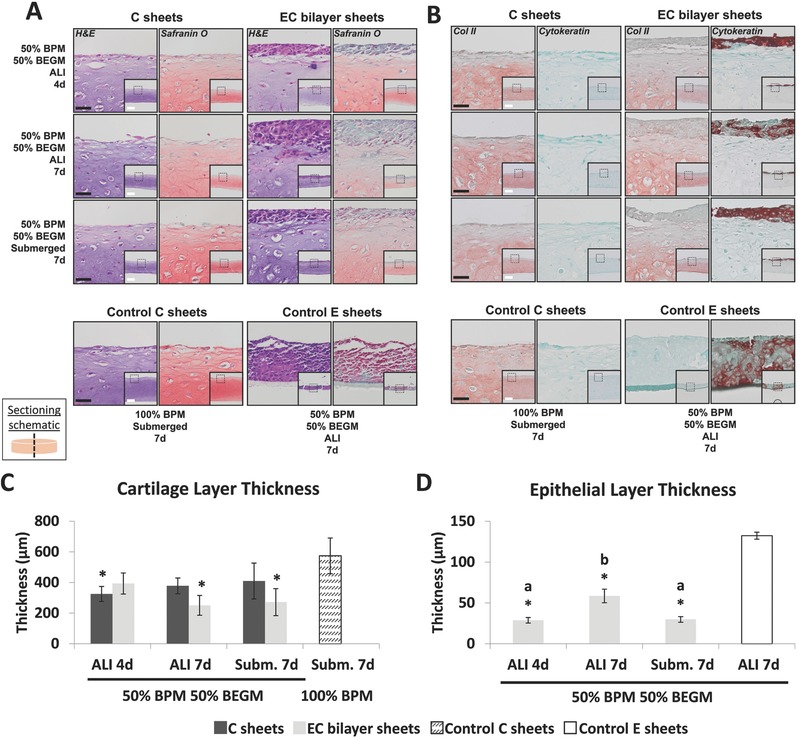
Histologic staining and thickness measurements of epithelialized cartilage sheets and controls. Epithelial–cartilage bilayer sheets and controls were stained with A) hematoxylin & eosin, Safranin O for GAG (pink/red) and B) antitype II collagen (red) and anticytokeratin (red) for epithelial cells. ALI, sheets cultured at air liquid interface for 4 or 7 d; submerged, sheets cultured submerged in media for 7 d. BPM, chondrogenic basal pellet media; BEGM, bronchial epithelial growth media. Fast green counterstain (blue/green). Black scale bars are 40 µm; white scale bars are 200 µm. Images without scale bars in a single row are the same magnification. C) Cartilage layer thickness and D) epithelial layer thickness were measured from histological images of cartilage (C) sheets (dark gray and patterned (control)), epithelial (E) sheets (white (control)) and epithelial–cartilage (EC) bilayers (light gray) (*N* = 3). ALI, sheets cultured at air–liquid interface for 4 or 7 d; Subm.; sheets cultured submerged in media for 7 d. BPM, chondrogenic basal pellet media; BEGM, bronchial epithelial growth media. *: significantly different compared to control cartilage sheets in (C) and control epithelial sheets in (D) (*p* < 0.05); a,b,c: Bilayer sheets without common letter differ (*p* < 0.05). Data shown as mean ± SD.

As a proof‐of‐principle of establishing an epithelial lining on the tissue engineered tracheas, epithelized cartilage (EC) bilayers were engineered by seeding hBECs on the surface of 2‐week‐old microsphere‐containing hMSC sheets. Bilayer tissues cultured at ALI, which is typically employed for respiratory epithelial culture, were compared to bilayers cultured submerged in media for 4 and 7 days (d). Hematoxylin and eosin (H&E) staining showed uniformly distributed chondrocytes within a homogeneous ECM that stained positive for GAG in the microsphere‐containing hMSC layer of all single and bilayer tissues (**Figure** **4**A). However, GAG staining appeared less intense in the cartilage layer of the EC bilayer sheets compared to cartilage only sheets (C sheets) alone in 50:50 basal pellet medium (BPM):bronchial epithelial growth medium (BEGM) and C control sheets in 100% BPM. Additionally, the cartilage layer of the epithelial–cartilage bilayers cultured at ALI showed a time‐dependent decrease in GAG staining from 4 to 7 d (Figure 4A).

Immunostaining for type II collagen was strong in all cartilage layers further confirming cartilaginous ECM synthesis (Figure 4B). Unlike GAG deposition, however, there were differences in the distribution of type II collagen between the experimental groups and the control C sheets in BPM. Control C sheets in BPM had more uniform type II collagen deposition throughout their thickness compared to C sheets in 50:50 BPM:BEGM and EC bilayer sheets, which had decreased type II collagen on the upper surface of the hMSC layers. Cartilage layers were negative for epithelium‐specific cytokeratin across groups. In contrast, localized cytokeratin staining was observed in all epithelial layers of EC bilayer sheets comparable to control E sheets.

#### 2.3.2 Thickness Quantification of Cartilage and Epithelial Components

To evaluate the quality of the epithelial and cartilage layers by another metric, the thickness of each tissue portion was quantified as a measure of spatial control over cell phenotype and tissue development. The cartilage layers of all C sheets and EC bilayer sheets cultured in the 50:50 mixture of BPM and BEGM were thinner compared to controls submerged in normal BPM, but differences were significant only for the C sheets ALI 4 d, EC bilayer ALI 7 d, and submerged 7 d groups (Figure 4C). Neither the ALI culture conditions nor duration significantly affected the thicknesses of hMSC layers in C sheets cultured in mixed media (Figure 4C). The cartilage layer of the EC bilayers showed a slight but not significant time‐dependent decrease in thickness from 4 to 7 d at ALI (Figure 4C). Moreover, all epithelial layers of EC bilayer sheets were significantly thinner compared to control E sheets cultured on cell culture inserts at ALI for 7 d (Figure 4D). Epithelial layers at ALI showed a significant time‐dependent increase in thickness from 4 d to 7 d (Figure 4D). Compared to ALI 7 d, E sheets submerged for 7 d were significantly thinner (Figure 4D).

### 2.4 Part III—Prevascular–Cartilage Composite Tubes and Tri‐Tissue Tracheas

#### 2.4.1 Macroscopic and Histological Assessment

**Figure 5:**
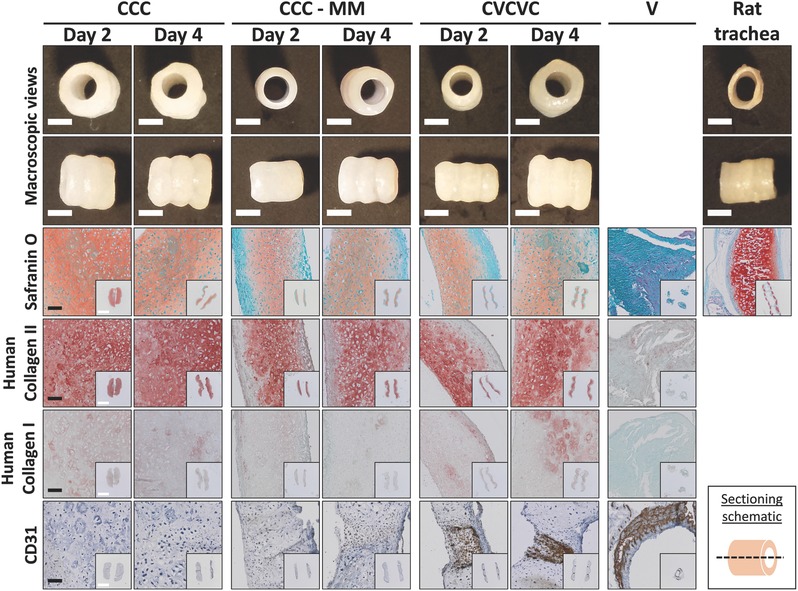
Macroscopic images and photomicrographs of Safranin O staining for GAG (pink/red), and human type II collagen (red), human type I collagen (red) and CD31 (brown) immunohistochemistry of prevascular–cartilage tissue engineered tubes and controls. Images and longitudinal sections of cartilage tubes in chondrogenic media (CCC), cartilage tubes in mixed chondrogenic and endothelial media (CCC–MM) and prevascular–cartilage tubes in mixed media (CVCVC) stacked on day 2 and day 4 after ring formation, and axial sections of prevascular rings (V) in endothelial media are depicted. Fast green counterstain (blue/green) for Safranin O, type I and II collagen; hematoxylin counterstain (blue) for CD31. Black scale bars are 200 µm; white scale bars are 2 mm. Images without scale bars in a single row are the same magnification.

While localized tissue differentiation and maturation are critical for the complex spatial organization of a trachea, coherent tissue fusion between the incorporated tissue types is also important for organ functionality. Timing may play a critical role in the success of tissue fusion^13^ as well as development of tissue‐specific phenotypes. As a result, cartilage and prevascular rings were cultured individually for varying time periods prior to stacking them for fusion into a composite tube to determine the impact of timing of fusion on resultant tissue structure and differentiation. With the goal to establish custom‐patterned, localized cartilage and prevascular soft tissue segments within the engineered tracheas, two prevascular rings (V) comprised of hMSCs and HUVECs were fused with three cartilaginous microsphere‐containing hMSC rings (C) in an alternating fashion to create prevascular soft tissue–cartilage composite tubes (CVCVC). Control cartilage‐only tubes were composed of three cartilage rings (CCC). Individual rings cultured for 2 or 4 d in their respective media were successfully fused into continuous tissue tubes. Tubes were harvested 2 weeks after cell seeding (**Figure** **5**; Figure S1, Supporting Information). Rings that were cultured individually for 6 d or longer did not fuse into tissue tubes.

All cartilage containing tubes had a pearly white surface and maintained an open lumen upon harvest. In the control cartilage‐only tubes that were cultured in mixed media (CCC‐MM; 50:50 BPM:endothelial growth medium‐2 (EGM‐2)) and the CVCVC groups but not in the cartilage‐only tubes cultured in 100% BPM (CCC), rings fused at day 4 resulted in qualitatively thicker‐walled constructs compared to rings fused at day 2. The walls of all constructs were grossly thicker than walls of rat tracheas. Cartilaginous components of all the tubes stained positively for GAG and type II collagen content with minimal type I collagen staining. Rat tracheal cartilage exhibited the strongest Safranin O staining. Remaining gelatin microspheres stained with type I collagen antibody, as expected. GAG and type II collagen staining in cartilage‐only tubes grown in BPM was the best distributed from the lumens to the outer tube edges whereas CCC‐MM and CVCVC tubes had noncartilaginous fibrous capsules on the outer surfaces.

CD31 staining (Figure 5; Figure S1, Supporting Information) showed that endothelial cells remained localized to the prevascular ring portions and some regions exhibited endothelial cell organization into prevascular structures. However, these structures were not as extensive as those in prevascular rings (V) grown for the duration of the experiment on glass tubes in EGM‐2, in which more of the endothelial cells were incorporated into prevascular structures. The majority of endothelial cells in prevascular rings grown in EGM‐2 were incorporated into complex prevascular plexuses. All cartilage only tubes, whether grown in BPM or mixed media, were negative for CD31 expression. Interestingly, more advanced plexus formation in the prevascular ring portion was noted in prevascular–cartilage composite tubes in another experiment (Figure S2, Supporting Information). Additionally, prevascular rings cultured in agarose wells for the duration of the experiment showed even more robust prevascular plexus formation (Figure S2, Supporting Information) compared to prevascular rings cultured on glass tubes (Figure 5).

#### 2.4.2 Biochemical Analysis

**Figure 6:**
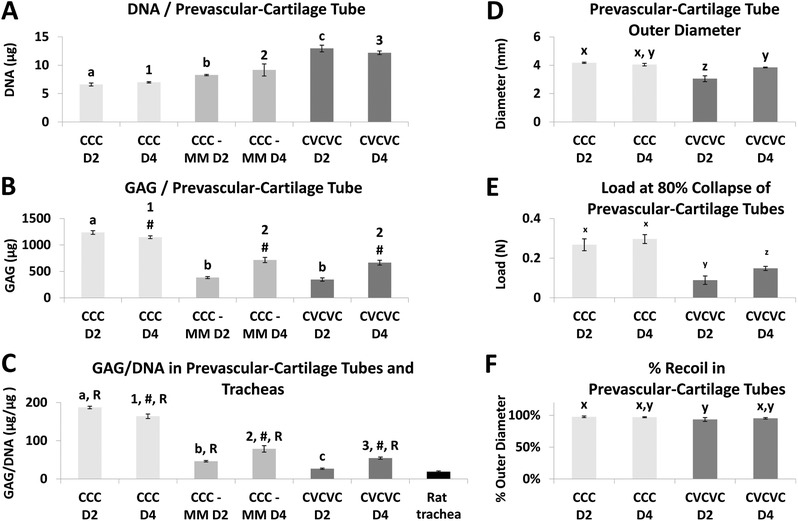
Biochemical and mechanical analysis of prevascular–cartilage tubes and controls. A) DNA, B) GAG and C) GAG normalized to DNA were acquired from tissue engineered cartilage only tubes in chondrogenic media (CCC, light gray, *N* = 3–4), cartilage tubes in mixed endothelial and chondrogenic media (CCC—MM, medium gray, *N* = 4) and prevascular–cartilage tubes in mixed media (CVCVC, dark gray, *N* = 4). C) Rat tracheal GAG/DNA was used for comparison (black, *N* = 4). D) Tube outer diameter, E) load required to collapse 80% of lumen diameter and F) tube recoil after luminal compression were measured in engineered tubes (*N* = 3). D2, Rings fused after 2 d of individual culture, D4, Rings fused after 4 d of individual culture. A–C) a,b,c: Day 2 groups without common letter differ (*p* < 0.05); 1,2,3: Day 4 groups without common number differ (*p* < 0.05); #: significantly different than Day 2 group (*p* < 0.05); R: significantly different than rat trachea. D–F) x,y,z: groups without common letter differ (*p* < 0.05). Data shown as mean ± SD.

The quality of the cartilage tissue in the composite tubes was also analyzed biochemically. CVCVC constructs, which were comprised of more cells, had significantly more DNA than cartilage‐only constructs (**Figure** **6**A). Surprisingly, CCC‐MM groups also had significantly more DNA than CCC tubes despite having the same number of incorporated cells. The GAG and GAG/DNA contents (Figure 6B,C) of constructs grown in BPM:EGM‐2 mixed media (CCC‐MM and CVCVC) was significantly less than those of CCC tubes grown in BPM, which corroborates histological findings (Figure 5). Additionally, cartilage‐only and CVCVC day 4 constructs in mixed media had significantly more GAG and GAG/DNA compared to day 2 constructs cultured in the same media. All engineered tubes except for CVCVC day 2 had significantly more GAG/DNA than native rat tracheas (Figure 6C).

#### 2.4.3 Mechanical Analysis

CCC and CVCVC tubes were mechanically characterized for luminal rigidity (Figure 6D–F). CVCVC day 2 tubes had the smallest outer diameter out of all the tubes analyzed (Figure 6D). CCC day 2, CCC day 4, and CVCVC day 4 tubes had outer diameters of ≈4 mm. Evaluation of tube mechanical properties showed that CVCVC tubes bore significantly less load at 80% luminal collapse compared to CCC tubes (Figure 6E). Additionally, CVCVC day 4 tubes bore significantly more load at 80% luminal collapse than CVCVC day 2 tubes. All engineered tubes recoiled to nearly 100% of the original diameter (Figure 6F).

#### 2.4.4 Tri‐Tissue Trachea Formation

**Figure 7:**
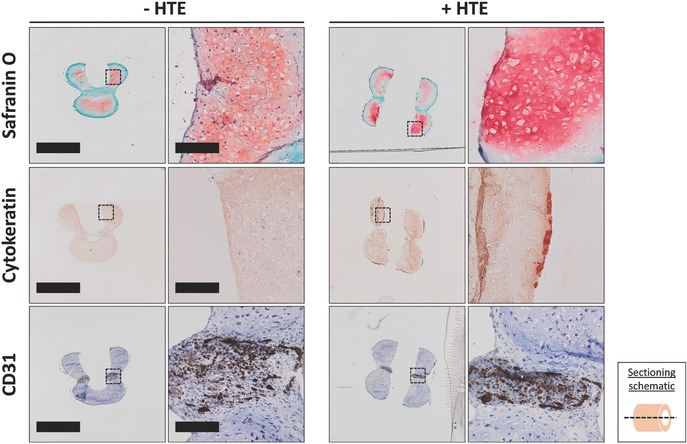
Histologic staining of tri‐tissue tracheas. CVC tubes were suspended in epithelial cell media in the absence (− HTE) or presence (+ HTE) of primary human tracheal epithelial (HTE) cells. Samples were harvested at 24 h, sectioned and stained with Safranin O (pink/red) with a fast green (blue/green) counterstain as an indication of cartilage extracellular matrix. To determine epithelial attachment, sections were stained for cytokeratin (red). Finally, sections were stained for CD31 (brown) and counterstained with Mayer's hematoxylin (blue) to identify endothelial cells. The left column scale bar for −HTE images is 2 mm and for the right column, it is 200 µm. Images in +HTE group are at same magnification as corresponding −HTE images. Dotted black box regions are shown in high magnification.

After fusing two cartilage rings and one vascular ring as before, CVC tubes were cultured in a suspension of human tracheal epithelial (HTE) cells for 24 h. Staining for GAG and CD31 demonstrated that cartilage matrix and endothelial prevascular structures, respectively, were still present after the additional culture in epithelial media (**Figure** **7**). Importantly, cytokeratin staining demonstrated the presence of adhered epithelial cells (Figure 7) to the surface of the CVC tubes.

#### 2.4.5 Subcutaneous Implantation in Athymic Mice

**Figure 8:**
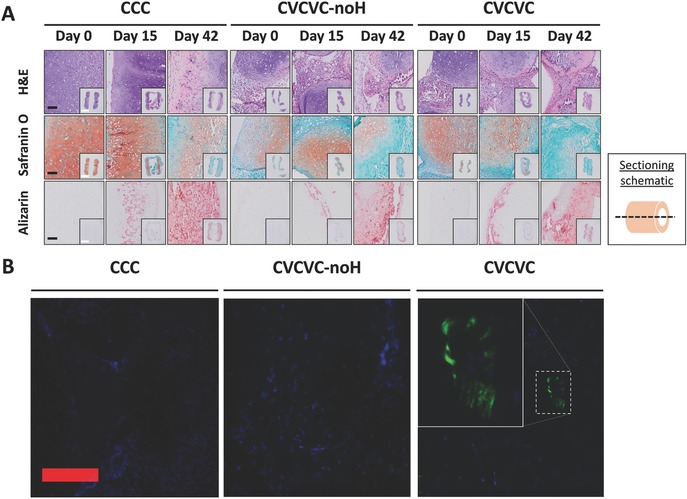
Histologic staining of tracheal tubes implanted subcutaneously in mice. Three types of tubes (CCC, CVCVC‐noH, and CVCVC) were implanted subcutaneously in mice for 0, 15, or 42 d. A) Longitudinal sections were stained with H&E, Safranin O (pink/red) with a Fast Green (blue/green) counterstain and alizarin red S (mineral is red). B) Tissue sections from day 42 samples were counterstained with DAPI and visualized for fluorescent FITC–UEA‐1 (green) perfusion staining. Black and red scale bars are 100 µm; white scale bars are 2 mm. Inset in (B) CVCVC group is 3× magnification of image.

After 19 d of in vitro culture, prevascular–cartilage composite tubes were implanted subcutaneously in athymic mice to assess vascularization of the engineered tissues. In addition to the CCC and CVCVC tubes described above, one additional group was included in this study. This new, third group consisted of three cartilage rings, initially cultured in BPM, separated by two vascular rings, composed only of MSCs, initially cultured in vasculogenic media. The vascular rings were seeded into the agarose molds and cultured like the vascular rings previously described but without the HUVECs. Upon fusing, these tubes were termed CVCVC‐noH and cultured in mixed media. Tissue engineered constructs that were fixed at day 0 before implantation and those harvested from the mice 15 and 42 d after implantation were processed for histology and immunohistochemistry (**Figure** **8**). The tubular structure was well maintained, and connective tissue had grown into the lumens of all samples. Although mechanical testing was not possible due to low sample numbers, explants were very firm to the touch and qualitatively stiffer than all in vitro‐cultured CVCVC or cartilage‐only tubes described previously. Composite neotracheas with intervening prevascular rings (with HUVECs) and control prevascular rings (without HUVECs) showed slightly more deformation from their original shape than tubes comprised solely of cartilage rings. The cartilage rings in composite tubes were slightly displaced with respect to each other along the longitudinal axis resulting in an offset tube. All samples showed the presence of GAG by Safranin O staining at day 0 and day 15 harvest, with CCC tubes exhibiting the strongest staining (Figure 8A). However, the staining for GAG was reduced in all samples at 42 d (*N* = 1). Alizarin red S staining demonstrated progressive calcium deposition within the samples over the course of the in vivo culture (Figure 8A). No calcium staining was seen at time 0. Frozen sections of day 42 explants were stained with DAPI and fluorescently imaged for perfused lectin staining of human endothelial cells to show human cell‐derived vasculature (Figure 8B) that anastomosed with the host. No lectin staining was seen in CCC and CVCVC‐noH samples. However, staining was seen in the CVCVC tube, and some HUVECs appeared to form structures with lumens.

## 3 Discussion

### 3.1 Part I—Cartilage Rings and Tubes with Defined Dimensions

Tracheal cartilage provides mechanical support to the airway, which is imperative for proper airway function. Our group has demonstrated that scaffold‐free, hMSCs with localized chondrogenic growth factor (TGF‐β1)‐delivering microspheres can be employed to engineer cartilaginous 2 mm inner diameter rings and tubes which are similarly sized to native rat tracheas.^8^ The microspheres promote chondrogenic differentiation of the stem cells through spatiotemporally controlled release of the growth factor.^14^ In this work, we hypothesized that the number of hMSCs and hMSCs with TGF‐β1‐laden microspheres used to fabricate 2 mm inner diameter rings can be varied to engineer rings with different wall thicknesses. At the same time, constructs with diameters >2 mm are required for testing in a larger animal model, like the rabbit (≈6 mm inner diameter^15^), and eventually translating for human use (≥12 mm inner diameter[[qv: 1a]]). The size of the custom culture wells and tube holders were modified to alter the diameter of resultant rings and tubes.

By changing the cell and microsphere numbers, cartilage ring (hMSC or hMSC + MS) thickness could be altered by 25–35%. However, even the thinnest rings were still not as thin as the rat trachea (Figure 2B). It is unclear if a thicker‐walled tracheal replacement will impede tracheal function in a rat airway defect model. Furthermore, the incorporation of additional tissue types and altered culture conditions, which will be necessary to functionalize the cartilage tube, will likely reduce the thickness of the cartilage. Finally, the customizable annular culture wells can easily be altered to obtain narrower troughs or modified trough geometry (e.g., U‐bottom vs. V‐bottom) to produce thinner rings or even other geometrical shapes if desired. In addition, the ultimate tensile stress (UTS, failure load normalized by loaded area) of all rings, except the 0.2 million cells per ring without microspheres condition, were similar among the groups as well as compared to the rat trachea, which means that engineered rings may fulfill the mechanical support requirement when used in rat airway repair.

Next, cartilaginous rings and tubes of larger diameters were engineered by modifying the cell culture wells and tube holders to produce 6 and 12 mm inner diameter constructs in addition to 2 mm constructs. Surprisingly, the number of hMSCs with microspheres required to reliably form complete tissue rings did not scale linearly with the circumference of the post. In fact, seeding 2.4 million hMSCs in 12 mm wells (6 × 0.4 million cells per ring) resulted in only 25% ring formation frequency. As a result, the cell number was increased by 25% in all sizes of rings (0.5 million per 2 mm ring, 1.5 million per 6 mm ring and 3 million per 12 mm ring) while keeping the microsphere‐to‐cell ratio constant. Still, the frequency of ring formation decreased with increasing diameter (Figure 3A). It is possible that the smaller curvature (1 per radius; 1 mm^−1^ for 2 mm, 0.33 mm^−1^ for 6 mm, 0.17 mm^−1^ for 12 mm) affected the ability of the hMSCs and microspheres to self‐assemble into continuous rings and ultimately elaborate chondrogenic matrix resulting in lower GAG/DNA and GAG/WW (Figure 3F,G). In fact, previous reports have shown that geometrical shape cues, like curvature, can direct hMSC differentiation.^16^ Nevertheless, chondrogenesis was not severely affected as GAG/DNA content only decreased by 23% and 25% for 6 mm and 12 mm tubes, respectively, compared to 2 mm tubes, while type II collagen staining remained strong. Tube wall thickness data indicate that 6 mm tubes (1.16 mm average thickness) may be of suitable size for the rabbit airway (0.97 mm average thickness) repair and 12 mm tubes (1.26 mm average thickness) may be adequately sized for use in humans whose tracheal cartilage is ≈1 mm thick.^17^ However, the 6 mm engineered cartilage tubes required only 8% of the rabbit tracheal load to collapse 80% of their respective lumen diameters and 12 mm cartilage tubes are likely substantially weaker than human tracheas (Figure 3I). As expected, the tube structural stiffness is proportional to the ratio of the radius to the wall thickness with greater ratios leading to more compliant tubes. Mechanical properties of engineered tracheal tubes containing multiple cell types were further weakened (Figure 6E). However, it is important to note that both the cartilage‐only tubes (Part I) and the prevascular–cartilage tubes (Part III) are immature constructs that were only grown for 3 and 2 weeks, respectively. So, it is not surprising that the neotracheas are weaker than their native counterparts, which had developed for much longer. In addition, it is unclear whether the weaker luminal collapse properties of the tracheal replacement would hinder airway function because healthy infant tracheas can safely collapse up to 50%, mostly by invagination of the posterior smooth muscle wall, during the first milliseconds of deep inspiration, while normal breathing results in minimal deformation.^18^ Importantly, all engineered tubes recoiled to nearly 100% of original diameter after luminal collapse just like the native rabbit trachea (Figure 3J). If necessary, mechanical properties of the tubes may be improved by increasing the wall thickness by using more hMSCs and microspheres or lengthening the culture time to elaborate more mature cartilaginous matrix. Taken together, these findings demonstrate the flexibility of the customizable culture system and show that cartilage tissue rings and tube with specific dimensions can be successfully engineered.

### 3.2 Part II—Epithelialized Cartilage Bilayers

Tracheal epithelium performs a crucial role in the innate host defense by (1) providing a protective physical barrier and (2) producing mucus to allow the body to clear infectious agents and environmental toxins.^19^ These functions can be temporarily or permanently compromised in patients with airway stenosis and malignant tumors or after tracheal resection.^20^ Hence, one of the main challenges for the translational application of tissue‐engineered tracheas is the ability to efficiently restore a functional epithelial cell layer. Isolated epithelial cells are typically cultured on protein‐coated cell culture inserts. Upon reaching confluence, the medium from inside the cell culture insert is removed, thereby exposing the epithelial sheet to air. This ALI culture model mimics the in vivo distribution of the airway lining; the apical side of epithelial cells faces the airway lumen and the basal side of the cells is attached to the basement membrane.[[qv: 9c,21]] We hypothesized that our established scaffold‐free, high‐cell density cartilaginous sheets^22^ may support epithelialization.

In proof‐of‐principle studies, we sought to engineer epithelial–cartilage bilayer sheet constructs. In this work, we were able to successfully engineer cohesive epithelial–cartilaginous bilayers. Qualitative histological analysis showed that while the 50:50 mixture of BPM and BEGM required for the maintenance of the two distinct cell types slightly reduced GAG content and altered type II collagen distribution in the underlying cartilaginous sheets relative to positive controls, the thickness of the epithelial layer seeded on top increased over time when exposed to air. Differences in GAG deposition in the C sheets compared to EC groups cultured in mixed media may be a result of hBEC–hMSC cell–cell communication, nutrient availability or diffusion limitations. Similarly, the E sheet positive control was thicker than the epithelial layers on the EC constructs, suggesting that nutrient availability, nutrient and oxygen diffusion or the presence of hMSCs altered epithelial layer development. Furthermore, expression of cytokeratins, an epithelial cell marker indicating structural cell integrity,^23^ was robust in the epithelial portion of the EC bilayer sheets and similar to that of the E control sheets cultured on standard cell culture inserts. The successful epithelial cell growth and differentiation at ALI and submerged in media achieved here is promising for engineering tubular tracheal replacements with a luminal epithelial coating.

### 3.3 Part III—Incorporation of Prevascular Rings into Tubes and Tri‐Tissue Tracheas

Similar to nearly all tissues in the body, native trachea has a vascular supply to facilitate oxygen and nutrient delivery and metabolic waste removal,^10^ critical for the survival of the investing soft tissue and epithelium lining the lumen.[[qv: 4g]] To develop a tissue engineered construct of clinically relevant size, the construct must be able to quickly develop a microvascular supply to survive after implantation. Neovascularization in replacement tracheas has been explored in only a few studies whose constructs contained vascular progenitor or endothelial cells obtained from adipose,^24^ bone marrow,^25^ or skin^26^ tissue. In vitro approaches to support vascularization of replacement tracheas have mostly relied on scaffold materials with potential angiogenic properties (e.g., decellularized allogeneic trachea^27^) or delivery of growth factors to drive angiogenesis.^28^ However, relying solely on host blood vessel infiltration may be insufficient in an orthotopic tracheal replacement because transplanted cells in the interior of the construct may die before becoming adequately microvascularized. Therefore, in this work, a prevascularization approach was developed to provide the framework for a microvasculature. By seeding endothelial cells with stromal supporting hMSCs in a ring mold and culturing for 2 weeks in EGM‐2, the endothelial cells were able to self‐assemble into prevascular structures (Figure 5) reminiscent of the prevascular cords and plexuses formed from blood islands in the mesenchymal condensations during embryonic vasculogenesis.^29^ Furthermore, with such a high cell number, the interior of these constructs may have been at low oxygen tension, a known provasculogenic stimulus,^30^ that may have enhanced prevascular structure formation.

The prevascular soft tissue rings were successfully stacked with cartilage rings resulting in ring fusion into tubes with similar architecture to native trachea (Figure 5). The presence of a prevascular network in the composite tubes will allow for the support of a luminal epithelium, a clear advance beyond cartilage only tubes. Unlike previous endothelialization approaches for tracheal tissue engineering, this strategy is advantageous because it avoids extensive material processing required for the use of decellularized materials,[[qv: 27a]] the need to reseed endothelial cells into the decellularized tissues,^25^ the need for ectopic implantation prior to orthotopic use^31^ and the use of angiogenesis‐inducing drugs.^32^ Additionally, it allows for precise spatial control over prevascular tissue generation.^33^

Stacked prevascular and cartilaginous rings fused together to form continuous multi‐tissue constructs. While the essential features of each tissue type were maintained throughout the coculture period, the resultant phenotype of each tissue type was diminished somewhat compared to that resulting from individual culture in specific medium for each respective tissue type. Prevascular rings cultured for the duration of the experiment in EGM‐2 showed a high degree of endothelial organization into prevascular plexus‐like structures (Figure 5). However, in composite tubes, there was a lower degree of endothelial cell incorporation into such structures, likely due to the decreased concentration of angiogenic growth factors in the mixed media. The GAG and GAG/DNA content in the prevascularized tubes cultured in mixed media was less than in tracheal tubes cultured in 100% BPM as demonstrated quantitatively by biochemical analysis (Figure 6A–C) and qualitatively by Safranin O staining (Figure 5). This decrease in GAG content was demonstrated to be, at least partially, due to the media and not the presence of prevascular rings because a similar decrease in GAG content was seen in cartilage‐only tubes cultured in the same 50:50 mixed media used with the prevascularized tubes. The weakened load bearing properties of prevascular tubes (Figure 6E) may be a result of the decreased GAG content from the culture in mixed media, which would decrease the mechanical properties of the engineered cartilage component.^34^ Nevertheless, tube recoil after luminal collapse testing was minimally affected (Figure 6F). To demonstrate the capability of this system to simultaneously support a further, third, physiologically relevant tracheal tissue type, CVC tubes were cultured in a suspension of epithelial cells to form tri‐tissue tracheal constructs. Importantly, the cartilage and prevascular phenotypes were maintained (Figure 7) through this additional period of culture while allowing for the attachment of epithelial cells on the surface of the CVC constructs (Figure 7). The epithelial cell coverage was not complete, but rather discontinuous. However, in areas of coverage, many cells were seen in juxtaposition with each other as opposed to individually attached cells, which may allow for the establishment of a continuous epithelium to provide an immunologic barrier function. These results demonstrate the feasibility of tri‐tissue tissue engineering in the current system.

The composite prevascular–cartilage tracheal constructs were then implanted subcutaneously in mice (Figure 7). Critical to their function of supporting an open airway, the engineered tracheas maintained their tubular architecture and their mechanical integrity was enhanced throughout in vivo culture. Substantial GAG staining was observed at the time of implantation and after 15 d in all groups. However, at the last time points, less GAG staining was evident. Progressive calcification of the constructs was noted (Figure 7A) with the staining appearing principally in the peripheral regions of the cartilage segments of the tubes. It may be that the chondrogenically driven hMSCs are contributing directly to tissue calcification themselves^35^ or by recruitment of host osteoprogenitor cells.^36^ It has been shown that control of hMSC fate is challenging as chondrogenically driven hMSCs can eventually lead to calcified tissue.^37^ It should be noted, however, that some calcification is seen in about half of native tracheas in the elderly without deleterious effects.^38^ Another key challenge upon implantation is host integration. If the constructs can hold a suture, then connecting them end‐to‐end via sewing to the healthy native tissue, potentially with a biocompatible sealant like fibrin glue, may be a simple and viable solution.

The intravenous injection of FITC–UEA‐1 allowed for the identification of human endothelial cells that were incorporated into perfused vasculature within the engineered neotracheas (Figure 7B). Predictably, no such staining was found in samples that lacked HUVECs. However, in the tracheal constructs with prevascular segments containing HUVECs, lectin‐labeled cells were seen in the explanted tissue. These positively staining cells were only seen in the prevascular segments of the tracheas indicating that there was no vascular invasion into the cartilage segments from the prevascular rings. The labeled endothelial cells were variably seen in circular or short cord‐like structures. The circular assemblies of stained HUVECs likely indicate patent lumens that were perfused with lectin containing host blood. While vascular lumen formation was not observed in vitro, the development of such mature, stable structures may have required the perfusion present in vivo.^39^ The spread HUVECs that do not enclose a lumen may be sections through the walls of vessels not oriented perpendicular to the plane of the section. Alternatively, as these stained, spread cells were often in the vicinity of lumen forming HUVECs, the vasculature formed by the transplanted cells may be leaky^40^ and as there are no lymphatics to drain the vascular filtrate, the lectin may simply be staining unincorporated HUVECs in the vicinity of the new HUVEC‐derived vasculature. To enhance vascularization in the future, it may be valuable to vary chondrogenic to vasculogenic media ratios during in vitro culture. In addition, microparticles presenting vasculogenic growth factors may be incorporated into prevascular rings, which may allow for a more uniformly distributed delivery of these factors than is possible by exogenous delivery alone to thicker constructs.

## 4 Conclusion

In this work, a modular, scaffold‐free approach for engineering complex tubular hollow organs was presented via the formation of a tri‐tissue engineered trachea. The three distinct tissues (i.e., cartilage, epithelial, and vascular) with defined spatial placement provide for luminal rigidity, a respiratory epithelium and prevascular structures to facilitate perfusion after implantation and anastomosis with host vasculature. Moving forward, prevascular–cartilaginous tubes may be epithelialized on the lumen with the aid of a tubular organ bioreactor.[[qv: 4d,f,41]] This modular, tubular tissue and organ engineering approach may also find great utility for regenerating other tissues such as large blood vessels and segments of the gastrointestinal (i.e., esophagus, intestines) and urinary (i.e., ureters) tract.

## 5 Experimental Section

*Experimental Design*: Four research objectives were examined in this body of work (Figure 1). The goal of Part Ia was to tune the thickness of engineered cartilage rings. Part Ib aimed to develop cartilage rings and tubes with custom‐defined lumen diameters. In Part II, a respiratory epithelium was engineered on the cartilaginous surface of cartilage tissues. Lastly, Part III focused on developing multi‐tissue type tubular constructs comprised of prevascular rings fused with cartilaginous rings, which were ultimately seeded with epithelial cells.

*Cell Culture*: Bone‐marrow‐derived hMSCs from a single donor were isolated using a Percoll gradient (Sigma‐Aldrich, St. Louis, MO) and the differential adhesion method, and then expanded in Dulbecco's modified Eagle's medium—low glucose (DMEM‐LG; Sigma‐Aldrich) containing 10% prescreened fetal bovine serum (Gibco Qualified FBS; Life Technologies, Carlsbad, CA or Sigma Premium FBS; Sigma‐Aldrich) and 10 ng mL^−1^ fibroblast growth factor‐2 (FGF‐2, R&D Systems, Minneapolis, MN) as previously described.^8^ Passage 2‐3 hMSCs were used in this study. BEAS‐2B human bronchial epithelial cells (hBECs) (ATCC; Manassas, VA) were cultured in bronchial epithelial cell growth medium (BEGM; Lonza, Walkersville, MD). Prior to use, culture flasks were coated overnight with 0.01 mg mL^−1^ fibronectin (Sigma‐Aldrich), 0.03 mg mL^−1^ type I collagen (Advanced BioMatrix, San Diego, CA), and 0.01 mg mL^−1^ bovine serum albumin (Thermo Fisher Scientific, Waltham, MA) at 37 °C. The next day, flasks were washed with PBS and hBECs were plated at 3.3 × 10^3^ cells cm^−2^ for expansion. Primary HUVECs (ATCC) were cultured in EGM‐2 (Lonza; Basel, Switzerland) and used at passage 3‐4. Human tracheal segments obtained at necropsy were stored at 4 °C in 50% Dulbecco's modified Eagle's medium‐high glucose (DMEM‐HG, Hyclone; South Logan, UT):50% Ham's F‐12 (Hyclone) supplemented with 2.5 × 10^−3^ m l‐glutamine (Sigma‐Aldrich), 5 µg mL^−1^ insulin (Sigma‐Aldrich), 5 µg mL^−1^ transferrin (Sigma‐Aldrich), 5 × 10^−6^ m hydrocortisone (Sigma‐Aldrich), and 2.5 µg mL^−1^ amphotericin (Sigma‐Aldrich). The tracheal segments were trimmed of excess connective and fatty tissue and treated with 0.1% protease XIV (Sigma‐Aldrich) at 4 °C for 16 h. The epithelial cells were isolated by gentle scraping of the luminal surface with a plastic coverslip. Isolated cell clumps were treated 5–7 min with Accutase (Sigma‐Aldrich) to dissociate. Cells were washed, resuspended in epithelial proliferation media (75% Ham's F‐12:25 % DMEM, supplemented with 5% FBS (Sigma‐Aldrich), 24 µg mL^−1^ adenine (Sigma‐Aldrich), 8.4 ng mL^−1^ cholera toxin (Sigma‐Aldrich), 10 ng mL^−1^ epidermal growth factor (Sigma‐Aldrich), 0.4 µg mL^−1^ hydrocortisone (Sigma‐Aldrich), 10 × 10^−6^ m Y‐27632 (Selleck Chemicals; Houston, TX), and 5 µg mL^−1^ insulin), and seeded onto a lawn of irradiated 3T3 fibroblasts. Media was changed daily and cultures were passaged 2–3 times (1:5) to produce 20–50 × 10^6^ epithelial cells. Epithelial cells were harvested for use by differential trypsinization to first remove irradiated 3T3 fibroblasts and then to collect the primary human tracheal epithelial cells.

*Microsphere Synthesis and Characterization*: Gelatin microspheres (11.1 w/v% Type A; Sigma‐Aldrich) were engineered as previously described.^22^ In this work, microspheres were crosslinked with 1 w/v% genipin (Wako Chemicals USA Inc., Richmond, VA) for 2.25–2.5 h. The percentage of crosslinked amine groups in the polymer was assessed by incubating microspheres for 1.75–2.75 min in ninhydrin solution.^8^ Experiments in this study used two batches of microspheres, which contained similar amounts of crosslinked amine groups in the polymer: 23.6 ± 4.7% and 25.7 ± 2.2%. The diameters of gelatin microspheres used in Parts I and III were 54.4 ± 40.4 µm (*N* = 354) and 43.3 ± 30.0 µm (*N* = 353) in Part II. For growth factor delivery, microspheres were loaded with 400 ng TGF‐β1 (PeproTech, Rocky Hill, NJ) per mg microspheres.

*Preparation of Custom Geometry Culture Wells*: Annular wells were machined as previously described[[qv: 13a]] or 3D printed (Objet 260 Connex, Stratasys) in three sizes: 2, 6, or 12 mm diameter posts surrounded by a 2 mm wide trough. Polydimethylsiloxane (PDMS; Sylgard 184, Dow Corning, Midland, MI) was cured in the molds and served as negative molds for casting 2% w/v agarose (Denville Scientific Inc., Metuchen, NJ) culture wells based on previously described methods.[[qv: 13a]] Prior to cell seeding, culture wells that were used to engineer cartilage rings were incubated overnight in chondrogenic BPM comprised of Dulbecco's modified Eagle's medium‐high glucose (DMEM‐HG; Sigma‐Aldrich), 1% ITS+ Premix (Corning Inc., Corning, NY), 10^−7^ m dexamethasone (MP Biomedicals, Solon, OH), 1 × 10^−3^ m sodium pyruvate (HyClone Laboratories), 100 × 10^−6^ m nonessential amino acids (Lonza Group, Basel, Switzerland), 37.5 µg mL^−1^ ascorbic acid‐2‐phosphate (Wako Chemicals USA Inc.) and 100 U mL^−1^ penicillin–streptomycin (Corning Inc.).^8^ Culture wells that were used to engineer prevascular rings were incubated in endothelial basal medium (EBM; Lonza Group).

*Assembly of Cartilage Rings and Tubes (Part I)*: In Part Ia, hMSCs (0.1 × 10^6^–0.4 × 10^6^ cells) with or without 0.75 mg TGF‐β1 laden microspheres/1 × 10^6^ cells in 50 µL media were seeded and cultured in 2 mm culture wells based on previously described methods.^8^ Rings with microspheres (“hMSC + MS”) were cultured in BPM and hMSC‐only groups (“hMSC”), which did not contain microspheres, were cultured in BPM supplemented with 10 ng mL^−1^ TGF‐β1. Tissue rings were grown in a humidified cell culture incubator at 37 °C and 5% CO_2_ for 21 d with media changes every 2 d.

In Part Ib, 0.5 × 10^6^ hMSCs, 1.5 × 10^6^ hMSCs, and 3 × 10^6^ hMSCs with 0.75 mg TGF‐β1‐loaded microspheres/1 × 10^6^ cells were seeded and cultured in 2, 6, and 12 mm culture wells in BPM, respectively. On day 2–3, cartilage rings were removed from the annular wells and stacked onto 2, 6, or 12 mm outer diameter borosilicate glass tubes (Adams & Chittenden Scientific Glass, Berkeley, CA) resulting in two‐ and five‐ring tissue tubes in each diameter. Tissue tubes were cultured horizontally on custom engineered polycarbonate holders in deep reservoirs (Axygen Scientific, Union City, CA) containing 100 mL BPM. Tissue tubes were grown in a humidified cell culture incubator at 37 °C and 5% CO_2_ for 21 d with media changes every 3 d.

*Assembly of Epithelial–Cartilage Bilayers (Part II)*: Cartilaginous sheets were formed by seeding 0.6 × 10^6^ cells with 0.75 mg TGF‐β1 laden microspheres/1 × 10^6^ cells onto cell culture inserts (6.5 mm diameter, 3 µm pore size; Corning). These were grown for 2 weeks with media changes (1 mL, BPM) every 2 d. Epithelial–cartilage bilayers were formed by seeding 8.3 × 10^4^ hBECs per sheet in 100 µL of BEGM on top of the hMSC+MS sheets at 2 weeks (“EC bilayer sheets”). Cartilage only sheets without an epithelial layer (“C sheets”) and epithelial only sheets without a cartilage layer (“Control E sheets”) served as controls. Control hBEC sheets were seeded on cell culture inserts (6.5 mm diameter, 0.4 µm pore size; Corning) previously coated overnight with 0.01 mg mL^−1^ fibronectin, 0.03 mg mL^−1^ type I collagen, and 0.01 mg mL^−1^ bovine serum albumin at 37 °C and rinsed with PBS. In all groups, seeded hBECs were allowed to settle and adhere for 3 d, after which the insert medium was removed in some of the groups to expose the constructs to an air–liquid interface (ALI) for 4 or 7 d (“ALI 4 d” and “ALI 7 d”). Some of the sheets remained submerged for 7 d (“Submerged 7 d”). Starting on Day 3 after hBEC seeding, all tissues were cultured in a 50:50 mixture of BPM and BEGM (“50% BPM 50% BEGM”) except cartilage control sheets which continued to be cultured in 100% BPM (“Control C sheets”). In summary, the experimental groups included: (1) Control C sheets (submerged; BPM; 7 d), (2) Control E sheets (ALI; 50% BPM 50% BEGM; 7 d), (3) C sheets (submerged; 50% BPM 50% BEGM; 7 d), (4) C sheets (ALI; 50% BPM 50% BEGM; 4 and 7 d), (5) EC bilayer sheets (submerged; 50% BPM 50% BEGM; 7 d), and (6) EC bilayer sheets (ALI; 50% BPM 50% BEGM; 4 and 7 d). Sheets were grown in a humidified cell culture incubator at 37 °C and 5% CO_2_ with media changes every 2 d.

*Assembly of Prevascular–Cartilage Composite Tubes and Tri‐Tissue Trachea Formation (Part III)*: 2 mm cartilage rings were assembled as in Part Ia using 0.4 × 10^6^ hMSCs and 0.3 mg TGF‐β1 laden microspheres. Prevascular rings were made from HUVECs (0.4 × 10^6^ cells) and hMSCs (0.4 × 10^6^ cells) suspended in 50 µL EGM‐2 and seeded in 2 mm ring‐shaped culture wells. On day 2, some cartilage and vascular rings were transferred onto 2 mm outer diameter glass tubes suspended in media. All cartilage rings and most prevascular rings that were transferred to glass tubes were used to make tubes on day 2 (“D2”) or day 4 (“D4”) after ring formation. Prevascular–cartilage tubes were formed by stacking three cartilage and two prevascular rings in an alternating sequence on day 2 or day 4 to make five‐ring tissue engineered tracheal tubes (“CVCVC D2” and “CVCVC D4”) and cultured in a 50:50 mixture of BPM and EGM‐2. As controls, at each stacking time point, three cartilage rings were stacked without intervening prevascular rings and cultured in BPM (“CCC D2” and “CCC D4”) or 50:50 BPM‐EGM‐2 mixed media (“CCC‐MM D2” and “CCC‐MM D4”). Some prevascular rings were cultured individually on glass tubes in EGM‐2 until the end of the experiment (“V”).

Alternatively, some prevascular rings composed of 0.2 × 10^6^ HUVECs and 0.2 × 10^6^ hMSCs were maintained in agarose molds for 14 d without being transferred to glass tubes on day 2 (“V–agarose”, Figure S2, Supporting Information). In an additional experiment, prevascular–cartilage tubes included two cartilage rings and one prevascular ring stacked at day 2 (“CVC D2”, Figure S2, Supporting Information). These were cultured in a 50:50 mix of BPM and EGM‐2 media. All tissue rings and tubes were grown in a humidified cell culture incubator at 37 °C and 5% CO_2_ for 15 d following ring formation with media changes every 2 d.

For tri‐tissue trachea formation, CVC tubes were formed as described earlier for CVCVC tubes, only with two cartilage rings and one vascular ring. These constructs were then placed in closed, perforated 1.5 mL microcentrifuge tubes containing a 0.5 mL suspension of 0.5 × 10^6^ HTE cells in epithelial proliferation medium and cultured on a rotisserie shaker (Barnstead Thermolyne, Dubuque, IA) for 24 h. Medium was replaced every 6 h.

*Tissue Harvest*: Cartilage rings and 2, 6, and 12 mm tubes were harvested 3 weeks after ring formation (Part I). Epithelial–cartilage bilayers and their cartilage‐only controls were harvested 7 and 10 d after bilayer creation, and epithelial‐only controls were harvested after 7 d of culture (Part II). Epithelial control sheets (no cartilage) were maintained on the cell culture insert membrane during tissue harvest and sectioning to limit damage to the thin sheets. Prevascular–cartilage tubes and their controls were harvested 15 d post cell seeding in ring molds, and tissue tubes to be seeded with epithelial cells were switched to an epithelial cell suspension for the final 24 h, 14 d post cell seeding in ring molds (Part III). All macroscopic images of tissues were taken with a Galaxy S4 phone camera (Samsung, Seoul, Korea). Tracheas from healthy rabbits (male New Zealand white rabbits, 9 months old (*N* = 6); Covance) and rats (male NIH Nude rats, 14–15 weeks old (*N* = 4); Taconic, Hudson, NY) were used for comparison. Use of harvested tissues from animals sacrificed for unrelated studies was approved by the Institutional Animal Care and Usage Committee (IACUC) at Case Western Reserve University (CWRU).

*Biochemical Analysis*: Tissue rings (*N* = 3–4 from Part Ia) and tubes (*N* = 4–5 two‐ring tissue tubes from Part Ib, *N* = 3–4 tubes from Part III and *N* = 4 rat tracheal segments) were digested in papain solution (Sigma‐Aldrich) at 65 °C.^42^ GAG and DNA contents were measured using dimethylmethylene blue (DMMB; Sigma‐Aldrich)^43^ and PicoGreen (Invitrogen, Carlsbad, CA) assays, respectively.^44^

*Histology and Immunohistochemistry*: Cartilaginous components of tissues from each Part (*N* ≥ 2) were evaluated for GAG via Safranin O (Acros Organics, Thermo Fisher Scientific) staining with a Fast Green (Fisher Chemical) counterstain and type II collagen deposition (ab34712 at 1:200 dilution; Abcam, Cambridge, UK) with a Fast Green counterstain as previously described.^8^ Sections of epithelial–cartilage bilayer sheets and their controls were stained with hematoxylin & eosin (H&E) (*N* = 3). Cytokeratin was detected using an anti‐pan‐cytokeratin antibody (sc‐81714 at 1:100 dilution; Santa Cruz Biotechnology, Santa Cruz, CA) with a Fast Green counterstain (*N* = 3). Type I collagen was visualized in prevascular–cartilage tissues using an anti‐type I collagen antibody (ab21287 at 1:250 dilution; Abcam) with a Fast Green counterstain (*N* = 2). In vitro‐cultured engineered prevascular–cartilage tubes and controls were stained for CD31, an endothelial cell marker, to evaluate prevascular structure formation (*N* = 2). Cryosectioned samples were stained using a CINtec Histology Staining Kit (Roche, Mannheim, Germany) and antihuman CD31 primary antibody (M0823 at 1:100 dilution; Dako, Carpinteria, CA) and counterstained with Mayer's hematoxylin (Thermo Fisher Scientific). The tri‐tissue tube (*N* = 1) was stained with Safranin O for cartilage, pan‐cytokeratin antibody for epithelial cells and CD31 antibody for endothelial cells. Stained tissue sections were imaged using an Olympus BX61VS microscope (Olympus, Center Valley, PA) with a Pike F‐505 camera (Allied Vision Technologies, Stadtroda, Germany).

*Cartilage and Epithelial Layer Thickness Analysis in Epithelial–Cartilage Bilayers*: Cartilage sheets, epithelial sheets, and epithelial–cartilage bilayers (*N* = 3 per group) stained with H&E were used to assess thickness. For quantification of the thickness of the cartilage portion in each cartilage sheet and epithelial–cartilage bilayer, a 10× magnified image of the center of each construct was acquired. The thickness of the cartilage portion was measured in three regions of interest (left, central and right) within this image. For quantification of the thickness of the epithelial portion within each epithelial control sheet and CE bilayer, 3 40× magnified images were acquired. The thickness of the epithelial portion was measured in three regions of interest (left, central, and right) within each image. The measurements were performed using ImageJ software (NIH, Washington, DC, USA).

*Tissue Dimension Measurements and Biomechanical Analysis*: Tissue engineered rings composed of different cell numbers (*N* = 3) and rat tracheas (*N* = 4) were analyzed using uniaxial tension to failure testing and the ring wall thicknesses were measured as previously described.^8^ Tissue engineered tubes (*N* = 3) and rabbit tracheas (*N* = 6) (Part Ib and III) were evaluated via luminal collapse and recoil as before with slight modifications.^8^ Freshly harvested five‐ring cartilaginous tubes of 2, 6, and 12 mm inner diameters and prevascular–cartilage 2 mm inner diameter tubes and their cartilage‐only controls were compressed by their respective luminal size at a rate of 0.5 mm min^−1^. Rabbit tracheas were evaluated in a similar manner by collapsing the lumen by each respective lumen diameter (5.17 ± 0.35 mm). Maximum load at 80% luminal collapse and the outer diameter after recoil were used for comparison. Engineered (2, 6, and 12 mm diameter) and native tracheal tube wall thicknesses were measured with calipers.

*Subcutaneous Implantation of Tracheal Constructs*: The surgical procedures used in this study were conducted according to a protocol approved by the IACUC of CWRU which adhered to the NIH Guide for the Care and Use of Laboratory Animals. Nine week old athymic mice (NCR nu/nu) from the CWRU Athymic Animal Facility were anesthetized using ketamine (160 mg kg^−1^)/xylazine (16 mg kg^−1^), and tracheal constructs were implanted subcutaneously on the dorsa of mice (3 or 4 constructs per mouse). The incisions were closed and the mice were administered 0.1 mg kg^−1^ buprenorphine at 0 and 12 h postsurgery.

Three different constructs were implanted: (1) tubes consisting of three cartilage rings prepared as above and cultured in BPM (CCC), (2) tubes consisting of three cartilage rings alternating with two prevascular rings prepared as above but with only hMSCs in the prevascular rings (without HUVECs) and cultured in 50:50 BPM:EGM‐2: (CVCVC‐noH), and (3) tubes consisting of three cartilage rings separated by two prevascular rings prepared as above and cultured in 50:50 BPM:EGM‐2 (CVCVC). All tubes were made by stacking rings at day 4 and culturing for a further 15 d prior to implantation. Tubes were collected on the day of implantation (*N* = 1 per group) and harvested on days 15 (*N* = 2 for CCC and *N* = 3 for CVCVC‐noH and CVCVC per group) and 42 (*N* = 1 per group) postimplantation. Prior to the 42 d harvest FITC‐UEA‐1 (Vector Labs, 100 µg in 100 µL of PBS) was perfused via tail vein injection to label human endothelial cells that were incorporated into perfused vasculature. Half of each tube was processed via paraffin sections which were stained with H&E, Safranin O, and alizarin red S (Sigma‐Aldrich). The other half of each tube was cryosectioned and sections were stained with DAPI (Thermo Fisher Scientific) to evaluate for fluorescent lectin staining. Bright‐field images were acquired as described earlier. Fluorescent images were taken on an Eclipse TE300 (Nikon, Tokyo, Japan) equipped with a Retiga‐SRV digital camera (Qimaging, Burnaby, BC, Canada).

*Statistical Analysis*: Statistical analysis of tissue engineered constructs and rat and rabbit tracheas was conducted using 1‐way ANOVA with Tukey's post hoc tests performed when *p* < 0.05 (InStat 3.06 software; GraphPad Software Inc., La Jolla, CA). Means of all values are reported with errors signifying standard deviation.

## Conflict of Interest

The authors declare no conflict of interest.

## Supporting information

Supplementary

Supplementary

Supplementary

Supplementary
