# Supplementary material for: A Modular Strategy to Engineer Complex Tissues and Organs
Source: Adv Sci (Weinh). 2018 Feb 14;5(5):1700402. doi: 10.1002/advs.201700402 (PMC5978945; doi:10.1002/advs.201700402)
Supplement: Supplementary file 1 — Supplementary [file ADVS-5-1700402-s002.pdf]

Copyright WILEY-VCH Verlag GmbH & Co. KGaA, 69469 Weinheim, Germany, 2017.

# Supporting Information

for *Adv. Sci.,* DOI: 10.1002/advs.201700402

# A Modular Strategy to Engineer Complex Tissues and Organs

*Anna D. Dikina, Daniel S. Alt, Samuel Herberg, Alexandra McMillan, Hannah A. Strobel, Zijie Zheng, Meng Cao, Bradley P. Lai, Oju Jeon, Victoria Ivy Petsinger, Calvin U. Cotton, Marsha W. Rolle, and Eben Alsberg**

Supporting Information

**Title A Modular Strategy to Engineer Complex Tubular Tissues and Organs**

*Anna D. Dikina†, Daniel S. Alt†, Samuel Herberg, Alexandra McMillan, Hannah A. Strobel, Zijie Zheng, Meng Cao, Bradley P. Lai, Oju Jeon, V. Ivy Petsinger, Calvin U. Cotton, Marsha W. Rolle, Eben Alsberg**

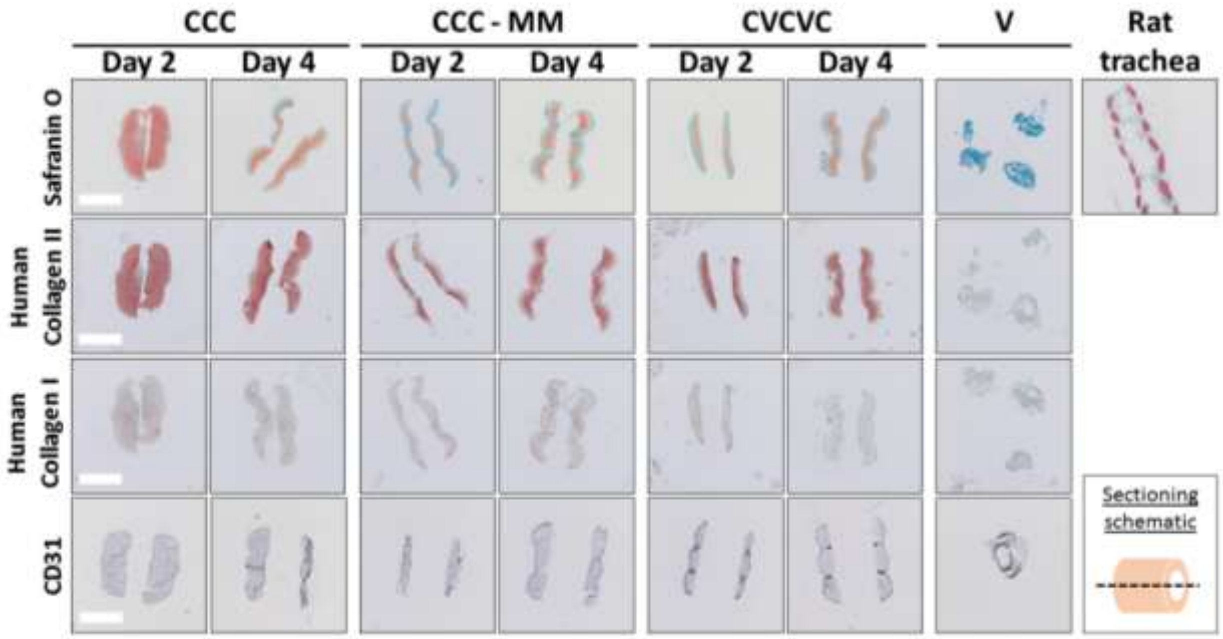


**Figure S1. Low magnification photomicrographs of Safranin O staining for GAG (pink/red), human type II collagen (red), human type I collagen (red), and CD31 (brown) immunohistochemistry of prevascular-cartilage tissue engineered tubes and controls.** Images of longitudinal sections of cartilage tubes in chondrogenic media (CCC), cartilage tubes in mixed chondrogenic and endothelial media (CCC – MM) and prevascular-cartilage tubes in mixed media (CVCVC) stacked on day 2 and day 4 after ring formation, and axial sections of prevascular rings (V) in endothelial media are depicted. Fast Green counterstain (blue/green) for Safranin O, type I and II collagen; hematoxylin counterstain (blue) for CD31. Scale bars are 2 mm. Images without scale bars in a single row are the same magnification.

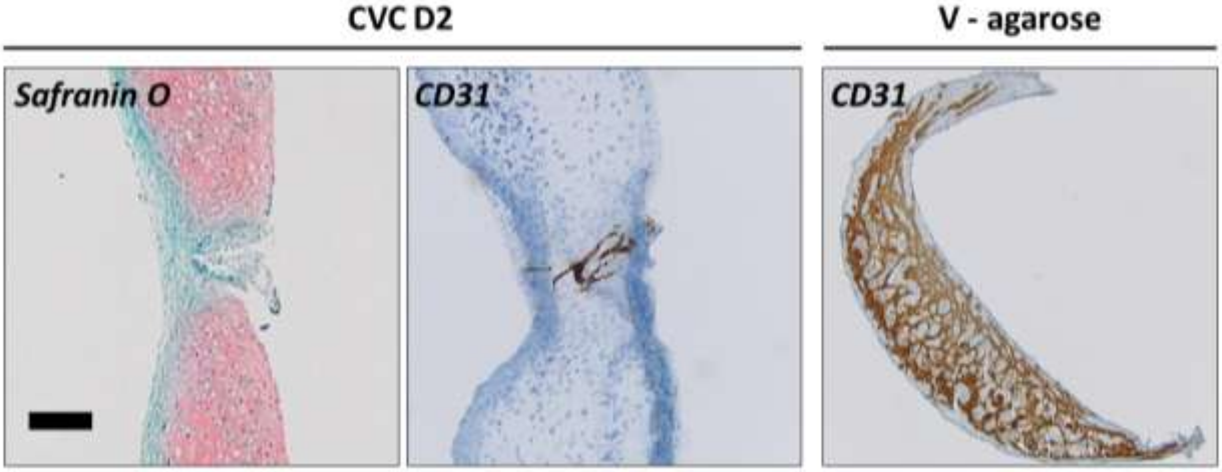


**Figure S2. Histological staining of prevascular-cartilage tubes and prevascular rings.** Safranin O (pink/red) for GAG and anti-CD31 (brown) staining of longitudinal sections of prevascular - cartilage composite tubes comprised of 2 cartilage rings and 1 prevascular ring (CVC D2) stacked on day 2 after ring formation and an axial section of a prevascular only ring (V - agarose) cultured in an agarose well for 14 days are shown. Fast Green counterstain (blue/green) for Safranin O and hematoxylin counterstain (blue) for CD31. Scale bar is 200 μm. Images without scale bars in a single row are the same magnification.

**Movie S1. Compressive mechanical testing of 2 mm inner diameter cartilage tube.** Tissue engineered tubes of 2 mm inner diameter were evaluated via luminal collapse and recoil. Freshly harvested 5-ring cartilaginous tubes were compressed by their respective luminal size at a rate of 0.5 mm / minute. Load at 80% luminal collapse and the outer diameter after recoil normalized to uncollapsed outer diameter were recorded. The video speed is 16x real time.

**Movie S2. Compressive mechanical testing of 6 mm inner diameter cartilage tube.** Tissue engineered tubes of 6 mm inner diameter were evaluated via luminal collapse and recoil. Freshly harvested 5-ring cartilaginous tubes were compressed by their respective luminal size at a rate of 0.5 mm / minute. Load at 80% luminal collapse and the outer diameter after recoil normalized to uncollapsed outer diameter were recorded. The video speed is 32x real time.

**Movie S3. Compressive mechanical testing of 12 mm inner diameter cartilage tube.** Tissue engineered tubes of 12 mm inner diameter were evaluated via luminal collapse and recoil. Freshly harvested 5-ring cartilaginous tubes were compressed by their respective luminal size at a rate of 0.5 mm / minute. Load at 80% luminal collapse and the outer diameter after recoil normalized to uncollapsed outer diameter were recorded. The video speed is 32x real time.
